# Higher rates of triple‐class virological failure in perinatally HIV‐infected teenagers compared with heterosexually infected young adults in Europe

**DOI:** 10.1111/hiv.12411

**Published:** 2016-09-14

**Authors:** A Judd, R Lodwick, A Noguera‐Julian, DM Gibb, K Butler, D Costagliola, C Sabin, A van Sighem, B Ledergerber, C Torti, A Mocroft, D Podzamczer, M Dorrucci, S De Wit, N Obel, F Dabis, A Cozzi‐Lepri, F García, NH Brockmeyer, J Warszawski, MI Gonzalez‐Tome, C Mussini, G Touloumi, R Zangerle, J Ghosn, A Castagna, G Fätkenheuer, C Stephan, L Meyer, MA Campbell, G Chene, A Phillips, The Pursuing Later Treatment Options II (PLATO II) Project Team for the Collaboration of Observational HIV Epidemiological Research Europe (COHERE) in EuroCoord, Murielle Mary Krause, Catherine Leport, Linda Wittkop, Peter Reiss, Ferdinand Wit, Maria Prins, Heiner Bucher, Diana Gibb, Julia Del Amo, Claire Thorne, Ole Kirk, Santiago Pérez‐Hoyos, Osamah Hamouda, Barbara Bartmeyer, Nikoloz Chkhartishvili, Andrea Antinori, Antonella d'Arminio Monforte, Luis Prieto, Pablo Rojo, Antoni Soriano‐Arandes, Manuel Battegay, Roger Kouyos, Pat Tookey, Jordi Casabona, Jose M. Miró, Deborah Konopnick, Tessa Goetghebuer, Anders Sönnerborg, Ramon Teira, Myriam Garrido, David Haerry, Dorthe Raben, Geneviève Chêne, Diana Barger, Christine Schwimmer, Monique Termote, Casper M. Frederiksen, Nina Friis‐Møller, Jesper Kjaer, Rikke Salbøl Brandt, Juan Berenguer, Julia Bohlius, Vincent Bouteloup, Mary‐Anne Davies, David Dunn, Matthias Egger, Hansjakob Furrer, Marguerite Guiguet, Sophie Grabar, Olivier Lambotte, Valériane Leroy, Sara Lodi, Sophie Matheron, Susana Monge, Fumiyo Nakagawa, Roger Paredes, Massimo Puoti, Michael Schomaker, Colette Smit, Jonathan Sterne, Rodolphe Thiebaut, Claire Thorne, Marc van der Valk, Natasha Wyss

**Affiliations:** ^1^ MRC Clinical Trials UnitUniversity College LondonLondonUK; ^2^ Department of Infection and Population HealthUniversity College LondonLondonUK; ^3^ Institut de Recerca Pediàtrica Hospital Sant Joan de DéuBarcelonaSpain; ^4^ Departament de PediatriaUniversitat de BarcelonaBarcelonaSpain; ^5^ CIBER de Epidemiología y Salud Pública CiberespBarcelonaSpain; ^6^ Department of Infectious Diseases and ImmunologyOur Lady's Children's HospitalCrumlin, DublinIreland; ^7^ INSERM, UPMC Univ Paris 06, Institut Pierre Louis d'épidémiologie et de Santé Publique (IPLESP UMRS 1136)Sorbonne UniversitésParisFrance; ^8^ Stichting HIV MonitoringAmsterdamThe Netherlands; ^9^ Division of Infectious Diseases and Hospital EpidemiologyUniversity of ZurichZurichSwitzerland; ^10^ Unit of Infectious and Tropical Diseases, Department of Medical and Surgical SciencesUniversity “Magna Graecia”CatanzaroItaly; ^11^ HIV and STD Unit, Infectious Disease ServiceHospital Universitari de Bellvitge. L'HospitaletBarcelonaSpain; ^12^ Istituto Superiore di SanitàRomeItaly; ^13^ Département of Infectious Diseases, Centre Hospitalier Saint‐PierreUniversité Libre de BruxellesBrusselsBelgium; ^14^ Department of Infectious DiseasesCopenhagen University Hospital, RigshospitaletCopenhagenDenmark; ^15^ INSERM U1219 – Centre Inserm Bordeaux Population HealthUniversité de BordeauxBordeauxFrance; ^16^ ISPED, Centre INSERM U1219‐Bordeaux Population HealthUniversité de BordeauxBordeauxFrance; ^17^ Clinical Microbiology Department, Complejo Hospitalario Universitario GranadaInstituto de Investigación Biosanitaria ibs.GranadaGranadaSpain; ^18^ Department of Dermatology, Venerology and Allergology, Center for Sexual Health and Medicine, St. Josef HospitalRuhr‐Universität BochumBochumGermany; ^19^ INSERM CESP U1018, AP‐HP Public Health DepartmentUniversité Paris‐Sud, Université Paris‐SaclayLe Kremlin‐Bicêtre ParisFrance; ^20^ HIV and Paeds Infectious Diseases DepartmentHospital 12 de OctubreMadridSpain; ^21^ Infectious Diseases ClinicsUniversity HospitalModenaItaly; ^22^ Department Hygiene, Epidemiology & Medical Statistics, Medical SchoolNational & Kapodistrian University of AthensAthensGreece; ^23^ Medical University InnsbruckInnsbruckAustria; ^24^ EA 7327, Faculté de Médecine site NeckerUniversité Paris Descartes, Sorbonne Paris CitéParisFrance; ^25^ APHP, Unité Fonctionnelle de Thérapeutique en Immuno‐InfectiologieHôpitaux Universitaires Paris Centre site Hôtel DieuParisFrance; ^26^ San Raffaele Scientific InstituteVita‐SaLute UniversityMilanItaly; ^27^ Department I of Internal MedicineUniversity Hospital of CologneCologneGermany; ^28^ Second Medical Department, Infectious Diseases UnitGoethe‐University HospitalFrankfurtGermany; ^29^ INSERM CESP U1018Université Paris‐Sud, Université Paris‐SaclayParisFrance; ^30^ AP‐HP Public Health DepartmentLe Kremlin‐BicêtreParisFrance; ^31^ Centre for Health and Infectious Disease ResearchUniversity of CopenhagenCopenhagenDenmark; ^32^ CHU de Bordeaux, Pole de sante publique, Service d'information medicaleBordeauxFrance

**Keywords:** Europe, perinatal HIV infection, virological failure, young people

## Abstract

**Objectives:**

The aim of the study was to determine the time to, and risk factors for, triple‐class virological failure (TCVF) across age groups for children and adolescents with perinatally acquired HIV infection and older adolescents and adults with heterosexually acquired HIV infection.

**Methods:**

We analysed individual patient data from cohorts in the Collaboration of Observational HIV Epidemiological Research Europe (COHERE). A total of 5972 participants starting antiretroviral therapy (ART) from 1998, aged < 20 years at the start of ART for those with perinatal infection and 15–29 years for those with heterosexual infection, with ART containing at least two nucleoside reverse transcriptase inhibitors (NRTIs) and a nonnucleoside reverse transcriptase inhibitor (NNRTI) or a boosted protease inhibitor (bPI), were followed from ART initiation until the most recent viral load (VL) measurement. Virological failure of a drug was defined as VL > 500 HIV‐1 RNA copies/mL despite ≥ 4 months of use. TCVF was defined as cumulative failure of two NRTIs, an NNRTI and a bPI.

**Results:**

The median number of weeks between diagnosis and the start of ART was higher in participants with perinatal HIV infection compared with participants with heterosexually acquired HIV infection overall [17 (interquartile range (IQR) 4–111) *vs*. 8 (IQR 2–38) weeks, respectively], and highest in perinatally infected participants aged 10–14 years [49 (IQR 9–267) weeks]. The cumulative proportion with TCVF 5 years after starting ART was 9.6% [95% confidence interval (CI) 7.0−12.3%] in participants with perinatally acquired infection and 4.7% (95% CI 3.9−5.5%) in participants with heterosexually acquired infection, and highest in perinatally infected participants aged 10–14 years when starting ART (27.7%; 95% CI 13.2−42.1%). Across all participants, significant predictors of TCVF were those with perinatal HIV aged 10–14 years, African origin, pre‐ART AIDS, NNRTI‐based initial regimens, higher pre‐ART viral load and lower pre‐ART CD4.

**Conclusions:**

The results suggest a beneficial effect of starting ART before adolescence, and starting young people on boosted PIs, to maximize treatment response during this transitional stage of development.

## Introduction

One of the major challenges for children and young people with perinatal HIV infection is the maintenance of long‐term adherence to treatment regimens to suppress virus and prevent resistant virus from developing 1. Paediatric HIV treatment regimens have relied heavily on drugs from one or more of the original three antiretroviral therapy (ART) classes [nucleoside or nucleotide reverse transcriptase inhibitors (NRTIs), non‐NRTIs (NNRTIs), and boosted protease inhibitors (bPIs)], as drugs from newer classes often lack paediatric pharmacokinetic data and are not approved for use in children (e.g. rilpivirine, elvitegravir in <18 years of age, dolutegravir in < 12 years of age), or do not have appropriate paediatric formulations, and are often expensive 2.

Current European guidelines recommend initiation of treatment in all infants aged < 1 year, in children ≥ 1 year of age according to age‐specific immunological thresholds (1–3 years, CD4 count ≤ 1000 cells/μL or CD4% ≤ 25%; 3–5 years, CD4 count ≤ 750 cells/μL or CD4% ≤ 25%; >5 years, CD4 count ≤ 350 cells/μL), and in any child with a World Health Organization (WHO) stage 3/4 or Centers for Disease Control and Prevention (CDC) category B/C event, largely to prevent progression to AIDS and death and to potentially optimize the ultimate CD4 count in adulthood 3. In a previous study, we showed how the risk of triple‐class virological failure (TCVF) of the three original drug classes in children also increased with older age at the start of ART 4. However, the main analysis of that study used a broad definition of TCVF which included now outdated treatment regimens, including unboosted PIs. A restricted analysis (including two NRTIs with either an NNRTI or a bPI) suggested that the overall rate of failure in children was over twice as high as in adults with heterosexually acquired HIV infection (hazard ratio 2.2; 95% confidence interval (CI) 1.6, 3.0; *P* < 0.0001). Here we further explored the difference in TCVF rates between perinatally infected children and young adults aged 15 to 29 years with heterosexual infection, and in particular looked at the effect of age at the start of ART on risk of TCVF.

## Methods

Data were pooled from 22 cohort studies participating in the Pursuing Later Treatment Options II (PLATO II) project in 2010, which is part of the Collaboration of Observational HIV Epidemiological Research Europe (COHERE) in EuroCoord (www.cohere.org and www.eurocoord.net), a collaboration of European cohort studies 5. This study included 22 cohort studies with data on heterosexually infected adults aged 15–29 years, and five cohort studies with paediatric data on perinatally infected children and young people aged < 20 years. We chose these age groups as we wished to compare perinatally HIV‐infected children and teenagers with adults with horizontal infection who were of a similar age group, and to show how the risk of TCVF differs across this age spectrum. A comparison of younger and older age groups has already been published elsewhere 6. The cohort studies included diagnosed HIV‐infected patients attending treatment services across Europe, collecting similar data items. Data submission and quality control procedures have been described previously 5.

Participants were antiretroviral‐naïve or had previous ART exposure for prevention of mother‐to‐child transmission (PMTCT), and started ART from 1998 onwards with an initial regimen of two or more NRTIs and either an NNRTI or a bPI. Follow‐up time began at the start of ART and data were censored at the last reported viral load measurement. Because the definition of virological failure used in this study required 4 months of use of a drug, children were only included if they had at least 4 months (122 days) of follow‐up. Time spent off ART (after ART had been started) was included as follow‐up. Participants were said to be off ART if they stopped all antiretroviral drugs. Treatment interruption was defined as cessation of all antiretrovirals for at least 7 days.

The main outcome measure was time to TCVF, defined as virological failure of at least two NRTI drugs, one NNRTI drug, and one bPI drug. Virological failure of a drug was defined as one viral load > 500 HIV‐1 RNA copies/mL following at least 4 months of continuous use of that drug, regardless of concomitant use of other drugs in that period. Sensitivity analyses were conducted using two variations of this virological failure definition: first, 6 months instead of 4 months of continuous drug use; secondly, viral load > 500 copies/mL after 4 months of continuous use had to be confirmed by a second viral load > 500 copies/mL while still on the drug or followed by discontinuation of the drug.

The date of the first HIV clinic visit was calculated as the earliest date of: first CD4 count, first viral load measurement, first visit, first diagnosis of an AIDS‐defining condition, or start of ART (including ART for PMTCT).

Kaplan−Meier and Cox regression methods were used to investigate the risk of TCVF after starting ART. Potential predictors of TCVF at the time of starting ART were sex, age group/risk group, region of origin, year of starting ART, initial regimen, previous ART exposure for PMTCT, pre‐ART AIDS diagnosis, CD4 (a binary measure combining CD4% <= 25% for patients <5 years of age and CD4 count <= 350 cells/μL for patients ≥5 years of age), and viral load (a binary measure, above or below the median viral load within each age group). In multivariable analyses, perinatally HIV‐infected young people aged 15–19 years at the start of ART were excluded because of their small number (*n* = 20) and lack of TCVF events. HIV‐related outcomes for those experiencing TCVF are described.

Analyses were performed using sas software, version 9.3 (SAS Inc., Cary, NC).

## Results

A total of 5972 participants fulfilling the inclusion criteria were identified, and their characteristics at the start of ART by risk group and age are summarized in Table 1. Overall, 806 participants (13%) had perinatally acquired HIV infection, and started ART aged < 20 years, and 5166 participants (87%) acquired HIV heterosexually and started ART aged 15–29 years. In the 15–19 years age category, 20 (7%) had perinatally acquired HIV infection and 264 (93%) had heterosexually acquired HIV infection, while all those < 15 years of age had perinatally acquired HIV infection, and all those ≥ 20 years of age had acquired HIV heterosexually. Initial regimens included lopinavir/r for 26% of participants with heterosexually acquired HIV infection and 17% of those with perinatally acquired HIV infection, efavirenz for 34% of those with heterosexually acquired HIV infection and 37% of those with perinatally acquired HIV infection, and nevirapine for 26% of those with heterosexually acquired HIV infection and 46% of those with perinatally acquired HIV infection.

**Table 1:** Characteristics of the 5972 included participants at the start of antiretroviral therapy (ART)

Characteristic	Risk group							
	Perinatal					Heterosexual		
	Age at start of ART (years)							
	< 2	2–4	5−9	10−14	15−19	15−19	20−24	25−29
Number (%) of participants	238 (4)	148 (2)	229 (4)	171 (3)	20 (<1)	264 (4)	1459 (24)	3443 (58)
Sex [*n* (%)]								
Male	101 (42)	83 (56)	119 (52)	79 (46)	6 (30)	58 (22)	319 (22)	946 (27)
Female	137 (58)	65 (44)	110 (48)	92 (54)	14 (70)	206 (78)	1140 (78)	2497 (73)
Region of origin [*n* (%)]								
Sub‐Saharan Africa	57 (24)	75 (51)	153 (67)	112 (65)	15 (75)	103 (39)	459 (31)	902 (26)
Europe	169 (71)	61 (41)	62 (27)	50 (29)	5 (25)	35 (13)	277 (19)	708 (21)
Other	11 (5)	12 (8)	13 (6)	8 (5)	0 (0)	6 (10)	195 (13)	536 (16)
Unknown	1 (<1)	0 (0)	1 (<1)	1 (1)	0 (0)	100 (38)	528 (36)	1297 (38)
Pre‐ART AIDS [*n* (%)]								
Yes	69 (29)	27 (18)	30 (13)	21 (12)	1 (5)	38 (14)	174 (12)	501 (15)
Time from diagnosis to ART start (weeks) [median (IQR)]	6 (2–18)	24 (5–102)	41 (5–185)	49 (9–267)	176 (55–404)	6 (1–19)	7 (2–33)	8 (2–44)
CD4 count								
Median (IQR) (cells/μL)	1170 (490–1818)	510 (279–793)	281 (160–482)	190 (94–286)	181 (89–231)	262 (163–395)	252 (155–375)	226 (125–325)
Missing [*n* (%)]	79 (33)	21 (14)	41 (18)	28 (16)	4 (20)	38 (14)	199 (14)	466 (14)
CD4 percentage								
Median (IQR)	25 (15–33)	14 (10–19)	13 (9–18)	12 (6–17)	–	–	–	–
Missing [*n* (%)]	81 (34)	20 (14)	48 (21)	31 (18)				
Viral load								
Median (IQR) (log_10_ copies/mL)	5.7 (5.0–5.9)	5.1 (4.7–5.6)	5.0 (4.6–5.3)	4.8 (4.2–5.2)	4.5 (4.2–4.8)	4.8 (3.9–5.2)	4.6 (4.0–5.0)	4.6 (4.0–5.1)
Missing [*n* (%)]	67 (28)	19 (13)	49 (21)	25 (15)	2 (10)	44 (17)	208 (14)	509 (15)
Year of starting ART [*n* (%)]								
1998–2000	43 (18)	31 (21)	28 (12)	15 (9)	2 (10)	33 (13)	232 (16)	565 (16)
2001–2003	81 (34)	51 (34)	81 (35)	43 (25)	1 (5)	90 (34)	416 (29)	1018 (30)
2004–2006	79 (33)	48 (32)	91 (40)	74 (43)	9 (45)	106 (40)	548 (38)	1261 (37)
2007–2009	35 (15)	18 (12)	29 (13)	39 (23)	8 (40)	35 (13)	263 (18)	599 (17)
Initial regimen [*n* (%)]								
NNRTI + ≥ 2 NRTIs	175 (74)	126 (85)	200 (87)	150 (88)	15 (75)	150 (57)	833 (57)	2107 (61)
bPI + ≥ 2 NRTIs	63 (26)	22 (15)	29 (13)	21 (12)	5 (25)	114 (43)	626 (43)	1336 (39)
PMTCT [*n* (%)]								
Yes	53 (22)	7 (5)	1 (< 1)	–	–	–	–	–

bPI, boosted protease inhibitor; IQR, interquartile range; NRTI, nucleoside reverse transcriptase inhibitor; NNRTI, nonnucleoside reverse transcriptase inhibitor; PMTCT, prevention of mother‐to‐child transmission.

Around half of participants with perinatally acquired HIV infection and three‐quarters of those with heterosexually acquired HIV infection were female, and the majority of most age groups were from sub‐Saharan Africa. In participants with perinatal HIV infection, the median age when first seen was 0.5 years [interquartile range (IQR) 0.0–2.7 years] for those born in Europe compared with 5.9 years (IQR 2.4–9.4 years) for those from sub‐Saharan Africa. The median duration between date first seen and the start of ART varied by risk group and age. It was around 6‐8 weeks for infants with perinatal HIV infection and participants who acquired HIV infection heterosexually, but much longer for children with perinatal HIV infection starting ART at age 5–9 years and 10–14 years [median 41 (IQR 5–185) and 49 (IQR 9–267) weeks, respectively], and highest in participants with perinatal HIV infection starting ART at age 15–19 years, although numbers were small [median 176 (IQR 55–404); *n* = 20] (Table S1).

Median CD4 cell count at the start of ART was lower in participants with perinatal HIV infection aged 10–14 and 15–19 years than in participants who acquired HIV heterosexually and also younger patients with perinatal HIV infection, and, as expected, the median CD4 percentage was higher in infants than in other perinatally infected groups. Similarly, viral load was highest in perinatally infected infants, and lowest at around 4.6 log_10_ copies/mL in all patients aged ≥ 10 years. Most participants started ART between 2000 and 2006. The vast majority (83%) of patients with perinatal HIV infection and 60% of those who acquired HIV heterosexually started on an NNRTI‐based regimen.

The overall median follow‐up from the start of ART was 3.5 (IQR 1.7–5.9) years. The median duration of follow‐up was markedly lower for participants with perinatal HIV infection aged 15–19 years [1.4 (IQR 1.0–2.3) years]. The rate (95% CI) of treatment interruption was 7.4 (6.5–8.4) per 100 person‐years in participants with perinatal HIV infection and 9.8 (9.4–10.3) per 100 person‐years in adults who had acquired HIV heterosexually. In those who had interrupted treatment, the rate (95% CI) of restarting was 67.4 (57.2–78.9) per 100 person‐years in participants with perinatal HIV infection and 56.4 (53.5–59.5) per 100 person‐years in those with heterosexually acquired HIV.

**Figure 1:**
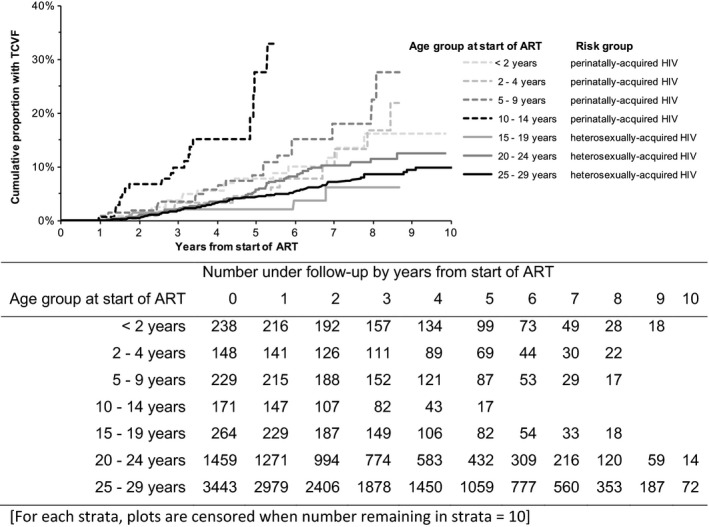
Kaplan−Meier plots showing the cumulative proportion of participants experiencing triple‐class virological failure (TCVF) by age at the start of antiretroviral therapy (ART) and risk group.

Just under half (357) of participants with perinatal HIV infection and 1774 of those who acquired HIV heterosexually experienced virological failure. By 5 years after starting ART, 49.9% (95% CI 45.8−54.0%) and 41.2% (95% CI 39.5−42.8%), respectively, had experienced virological failure, with no marked differences by age group (Table S1). By the end of follow‐up, 216 of 806 patients with perinatal HIV infection and 1539 of 5166 patients with heterosexually acquired HIV had started a third drug class, and 70 with perinatal HIV infection and 182 with heterosexually acquired HIV had developed TCVF. The cumulative proportion of participants experiencing TCVF by age at the start of ART and risk group is shown in Table 2 and Figure 1, and was higher in patients with perinatal HIV infection compared with those with heterosexually acquired HIV overall, and was particularly high in perinatally infected participants aged 10–14 years, at 27.7% (95% CI 13.2–42.1) by 5 years (Table S1). Of the 252 patients overall with TCVF, 12 (4.8%) had never attained a viral load ≤ 500 copies/mL.

**Table 2:** Participants developing triple‐class virological failure (TCVF): number, proportion, and predictors from univariable and multivariable Cox models

	Estimated cumulative proportion developing TCVF by 5 years (%)^b^	Univariable results			Multivariable results		
		HR	95% CI	*P*‐value	HR	95% CI	*P*‐value
Sex							
Male	6.9 (5.2–8.5)	1	–	0.0055	1	–	0.17
Female	4.9 (3.9–5.8)	0.70	0.54–0.90		0.83	0.64–1.09	
Age at start of ART and risk group							
< 2 years; perinatally acquired	7.8 (3.7–12.0)	1.83	1.11–3.00	<0.0001	2.16	1.25–3.72	<0.0001
2–4 years; perinatally acquired	4.5 (0.6–8.5)	1.67	0.90–3.09		1.45	0.76–2.75	
5–9 years; perinatally acquired	8.4 (3.9–12.9)	2.49	1.56–3.96		1.96	1.18–3.25	
10–14 years; perinatally acquired	27.7 (13.2–42.1)	6.16	3.81–9.97		5.19	3.11–8.65	
15–19 years; heterosexually acquired^a^	2.1 (0.0–4.1)	0.84	0.39–1.80		0.76	0.36–1.65	
20–24 years; heterosexually acquired	5.8 (4.0–7.6)	1.34	0.98–1.82		1.36	0.99–1.85	
25–29 years; heterosexually acquired	4.5 (3.5–5.4)	1	–		1	–	
Region of origin							
Sub‐Saharan Africa	6.8 (5.2–8.5)	1	–	0.0003	1	–	0.012
Europe	4.5 (3.0–6.0)	0.56	0.40–0.79		0.59	0.41–0.84	
Other/unknown	5.1 (3.9–6.3)	0.61	0.46–0.81		0.79	0.58–1.08	
Pre‐ART AIDS							
No	4.9 (4.0–5.7)	1		0.0044	1	–	0.039
Yes	8.5 (6.1–11.0)	1.55	1.15–2.08		1.38	1.02–1.88	
Initial regimen							
NNRTI + ≥2 NRTIs	6.1 (5.1–7.1)	1	–	0.0011	1	–	0.0023
bPI + ≥2 NRTIs	3.9 (2.5–5.3)	0.58	0.42–0.80		0.59	0.42–0.83	
Pre‐ART CD4 composite measure^c^							
CD4% < 25%/CD4 < 350 cells/μL	6.3 (5.2–7.4)	1	–	0.044	1	–	0.009
CD4% ≥ 25%/CD4 ≥ 350 cells/μL	3.8 (2.3–5.3)	0.74	0.53–1.03		0.85	0.60–1.20	
Unknown	4.4 (2.6–6.1)	0.68	0.47–0.99		0.48	0.29–0.77	
Pre‐ART viral load							
Below median for age group	3.3 (2.3–4.3)	1	–	0.0018	1	–	0.0005
Above median for age group	7.0 (5.6–8.3)	1.67	1.26–2.22		1.66	1.24–2.22	
Unknown	6.6 (4.4–8.7)	1.47	1.01–2.14		2.13	1.34–3.40	

ART, antiretroviral therapy; bPI, boosted protease inhibitor; CI, confidence interval; HR, hazard ratio; IQR, interquartile range; NRTI, nucleoside reverse transcriptase inhibitor; NNRTI, nonnucleoside reverse transcriptase inhibitor.

Year of starting ART (unadjusted *P* = 0.43), previous ART exposure for prevention of mother‐to‐child transmission (unadjusted *P* = 0.083) and sex (adjusted *P* = 0.09) were all not significant.

a For the age group 15–19 years, data are shown for participants with heterosexually acquired HIV infection only, as there were only 20 participants and no events in the 15‐19‐year age group for participants with perinatally acquired HIV infection.

b Kaplan−Meier estimates (95% confidence intervals).

c CD4% for < 5 years; CD4 count for ≥ 5 years.

In a multivariable model (Table 2), after adjustment for other factors, there was a strong association between age group and risk group and risk of TCVF. Participants with perinatal HIV infection aged 10–14 years at ART initiation had a markedly raised risk of failure [hazard ratio (HR) 5.19; 95% CI 3.11–8.65], participants with perinatal HIV infection aged < 10 years had an increased risk (e.g. 5–9 years: HR 1.96; 95% CI 1.18–3.25), and those with heterosexually acquired HIV aged 15–19 years (HR 0.76; 95% CI 0.36–1.65) and 20–24 years (HR 1.36; 95% CI 0.99–1.85) had a similar risk, compared with those with heterosexually acquired HIV infection aged 25–29 years. Additionally, participants originating from Europe compared with sub‐Saharan Africa and those starting bPI regimens compared with NNRTI regimens had a lower risk, and those with an AIDS diagnosis before starting ART had a higher risk, as did those with a higher pre‐ART viral load and lower pre‐ART CD4. There was no difference in the risk of failure by sex, ART exposure for PMTCT in infancy, or year of ART initiation. Sensitivity analyses conducted using two variations of the virological failure definition produced similar results, including in patients < 2 years of age with perinatal infection.

Clinical outcomes following TCVF are shown in Table S2. Overall, 1 year after TCVF, CD4 counts were higher in younger participants with perinatally acquired HIV infection and those with heterosexually acquired HIV infection than in participants with perinatal HIV infection aged 10–14 years, although numbers were small. Similarly, median viral load was higher in perinatally infected participants aged 10–14 years and lower in all other participant groups. Five per cent (three of 64) of participants with perinatal HIV infection with follow‐up data available after TCVF had been diagnosed with AIDS or died, compared with 11% (19 of 170) of participants with heterosexually acquired HIV infection; 9% (six of 64) of perinatally infected participants compared with 25% (43 of 170) of those with heterosexually acquired HIV infection had started at least one new drug by 1 year following TCVF.

## Discussion

In this large collaborative study in which the risk of TCVF from the start of ART in perinatally HIV‐infected children was directly compared with that in adults with heterosexually acquired HIV infection, 4.2% developed TCVF overall. The highest risk of TCVF was in 10‐14‐year‐old participants with perinatal infection; for this group, the cumulative incidence of TCVF by 3 years was nearly 10%, rising sharply to just under 30% by 5 years, while in all other age groups the proportion at 5 years was < 9%. This trend was shown in the multivariable analysis in which the rate of failure in this group was five times higher than in adults aged 25–29 years with heterosexually transmitted HIV infection. These results augment those of our earlier study which described the rate and predictors of TCVF in children, and which presented only a very simple analysis comparing rates between children and adults 4. Here, we estimated the differing rates of TCVF across the age spectrum from infants to young adults, as well as by transmission group.

Participants with perinatally acquired HIV infection aged 10–14 years had not only the highest rate of TCVF but also the longest delay in starting ART following HIV diagnosis, and the lowest CD4 counts at the start of ART. Many of these young people were born in sub‐Saharan Africa, some without access to antenatal testing; they may have complex family issues with a high proportion being orphans, and HIV symptoms may not have been recognized, leading to delayed diagnosis 7, 8. Some will have insecure immigration status which can intensify concerns about HIV diagnosis and treatment, and disclosure of HIV infection in the family 9, 10. There can also be issues of coming to terms with their own HIV status, secrecy and guilt among other family members, and dealing with HIV infection in the context of the adolescent transitional stage of development 11, 12. Once ART has been started, studies have found lower rates of viral suppression in young people with perinatal HIV infection compared with adults 13, which are probably attributable to adherence issues and poor clinic attendance rather than drug efficacy, both common problems among young adults living with other chronic diseases 1, 14, 15, 16. Findings from this study support the benefit of earlier age at diagnosis where possible, and initiation of ART prior to the adolescent period, which will probably also favour improved immunological health 17.

In this study, most participants with perinatal HIV infection started on an NNRTI‐based regimen, while two‐fifths of participants with heterosexually acquired infection in the study started on bPIs, and those starting with bPI‐based regimens were less likely to develop TCVF. In our previous study with resistance test data, we found that most children on an NNRTI regimen had resistance mutations, but PI resistance was not seen 4. Resistance results from the PENPACT‐1 trial showed that delaying switching for those starting a PI‐based regimen was a reasonable strategy, but that more rapid switching from an NNRTI‐based regimen following virological failure was needed to avoid accumulation of NRTI resistance mutations 18; in this study, these findings would mean that a higher proportion of the NNRTI group would be at risk of TCVF. However, results from a large adult trial of second‐line treatment following NNRTI‐based first‐line therapy suggested that participants given a PI as second‐line therapy retained NRTI virological activity without evidence of increased toxicity, even in the presence of NRTI resistance mutations 19, suggesting that, although in our study participants met the criteria of TCVF, its impact may not be substantial.

European guidelines now recommend that if adherence is questionable, as is often seen in teenagers, a PI‐based regimen should be initiated because of its higher genetic barrier to resistance 3, 20. Many of the participants with perinatal HIV infection on PI‐based regimens in our study would have been on lopinavir/r regimens, which are unpopular among adolescents as they require twice‐daily administration, but now both darunavir and atazanavir are available to adolescents in once‐daily formulations and have better tolerability. Furthermore, two new once‐daily single‐tablet regimens combining integrase inhibitors with an NRTI backbone have recently been licensed for adults and children aged 12 years and older weighing at least 35–40 kg and may become a suitable alternative to PI‐based regimens for adolescents 21, 22, 23, 24, 25. Similarly, innovative treatment strategies designed to improve adherence are needed for adolescents, and the phase II BREaks in Adolescent and child THerapy using Efavirenz and two nRtis (BREATHER) trial recently reported noninferiority of virological suppression in young people on efavirenz plus two NRTIs having weekends off compared with continuous therapy; long‐term follow‐up of these trial participants has begun 26.

In conclusion, for participants with perinatal HIV infection, these findings indicate the need to diagnose and start ART earlier in childhood, and adopt a strategic approach in ART selection to prevent the emergence of resistance. These measures may help children and adolescents achieve and sustain virological suppression as they approach adulthood.

## Conflict of interest

B Ledergerber has received travel grants, grants or honoraria from Janssen, Gilead and ViiV. A Mocroft has received honoraria, consultancy fees, travel support and/or lecture fees from Gilead, BMS, Pfizer, Merck, BI and Wragge LLC. A Phillips has received consultancy/ payment for lectures from Gilead, GSK Biologicals, Abbvie. D Costagliola was a member of the French Gilead HIV board up to 2015. In the past 3 years she gave lectures for Janssen‐Cilag, Merck‐Sharp & Dohme‐Chibret, ViiV and received travel/accommodations/meeting expenses from Gilead, ViiV, Janssen‐Cilag. She conducted post‐marketing studies for Janssen‐Cilag, Merck‐Sharp & Dohme‐Chibret and ViiV. She is currently a consultant of Innavirvax. Norbert H. Brockmeyer received grants and travel/accommodations/meeting expenses from Gilead, Sanofi Pasteur, Roche, AbbVie, BMS and Janssen Cilag, and payments to his institution for consultancy and development of educational presentations from Gilead and for consultancy for MSD. Maria Isabel Gonzalez‐Tome has received Gilead fellowship 2014 and has collaborated in clinical trials (Gilead, Pfizer.)

## Author contributions

All members of the PLATO II Writing Group participated in discussions on the design of the study, the choice of statistical analyses and interpretation of the findings, and were involved in the preparation and review of the final manuscript for submission. In addition, Rebecca Lodwick and Andrew Phillips were responsible for performing all analyses; Dr Lodwick acts as guarantor for the analyses and has full access to the data set. Study concept and design: A. Judd, R. Lodwick, A. Noguera‐Julian, D. M. Gibb, K. Butler and A. Phillips. Acquisition of data: A. Judd, A. Noguera‐Julian, D. M. Gibb, K. Butler, D. Costagliola, C. Sabin, A. van Sighem, B. Ledergerber, C. Torti, A. Mocroft, D. Podzamczer, M. Dorrucci, S. De Wit, N. Obel, F. Dabis, A. Cozzi‐Lepri, F. García, N. Brockmeyer, J. Warszawski, M. Gonzalez‐Tome, C. Mussini, G. Touloumi, R. Zangerle, J. Ghosn, A. Castagna, G. Fätkenheuer, C. Stephan, L. Meyer, M. A. Campbell, G. Chene and A. Phillips. Analysis and interpretation of data: A. Judd, R. Lodwick, M. Gonzalez‐Tome, A. Noguera‐Julian, D. M. Gibb, K. Butler and A. Phillips. Drafting of the manuscript: A. Judd, R. Lodwick, D. M. Gibb and A. Phillips. Critical revision of the manuscript for important intellectual content: all authors.

## Supporting information


**Table S1.** Number and proportion of participants developing virological failure, interrupting treatment, and developing TCVF, by risk group and age at the start of ART.
**Table S2.** Outcomes of TCVF, by risk group and age at the start of ART.

## Appendix 1 authors/writing group (affiliation)

Ali Judd (AALPHI, CHIPS), Rebecca Lodwick (statistician), Antoni Noguera‐Julian (CORISPE‐cat), D. M. Gibb (CHIPS), Karina Butler (CHIPS), Dominique Costagliola (ANRS CO4 FHDH), Caroline Sabin (UK CHIC), Ard van Sighem (ATHENA), Bruno Ledergerber (SHCS), Carlo Torti (MODENA), Amanda Mocroft (EUROSIDA), Daniel Podzamczer (PISCIS), Maria Dorrucci (CASCADE), Stéphane De Wit (St Pierre Cohort), Niels Obel (Danish HIV Cohort), François Dabis (ANRS CO3 AQUITAINE), Alessandro Cozzi‐Lepri (ICONA), Federico García (Co‐RIS), Norbert Brockmeyer (KOMPNET), Josiane Warszawski (ANRS CO1 EPF), Maribel Gonzalez‐Tome (CORISPES‐Madrid Cohort), Cristina Mussini (MODENA), Giota Touloumi (AMACS), Robert Zangerle (AHIVCOS), Jade Ghosn (ANRS CO6 PRIMO), Antonella Castagna (San Raffaele), Gerd Fätkenheuer (Cologne‐Bonn), Christoph Stephan (FRANKFURT), Laurence Meyer (ANRS CO2 SEROCO), Maria Athena Campbell (Copenhagen RCC representative), Genevieve Chene (Bordeaux RCC representative) and Andrew Phillips (PLATO II project leader; UK CHIC).

## Appendix 2 COHERE committees, coordinating centres, project leads and statisticians

Steering Committee ‐ Contributing Cohorts: Robert Zangerle (AHIVCOS), Giota Touloumi (AMACS), Josiane Warszawski (ANRS CO1 EPF/ANRS CO11 OBSERVATOIRE EPF), Laurence Meyer (ANRS CO2 SEROCO), François Dabis (ANRS CO3 AQUITAINE), Murielle Mary Krause (ANRS CO4 FHDH), Jade Ghosn (ANRS CO6 PRIMO), Catherine Leport (ANRS CO8 COPILOTE), Linda Wittkop (ANRS CO13 HEPAVIH), Peter Reiss (ATHENA), Ferdinand Wit (ATHENA), Maria Prins (CASCADE), Heiner Bucher (CASCADE), Diana M. Gibb (CHIPS), Gerd Fätkenheuer (Cologne‐Bonn), Julia del Amo (CoRIS), Niels Obel (Danish HIV Cohort), Claire Thorne (ECS), Amanda Mocroft (EuroSIDA), Ole Kirk (EuroSIDA), Christoph Stephan (Frankfurt), Santiago Pérez‐Hoyos (GEMES‐Haemo), Osamah Hamouda (German ClinSurv), Barbara Bartmeyer (German ClinSurv), Nikoloz Chkhartishvili (Georgian National HIV/AIDS), Antoni Noguera‐Julian (CORISPE‐cat), Andrea Antinori (ICC), Antonella d'Arminio Monforte (ICONA), Norbert Brockmeyer (KOMPNET), Luis Prieto (Madrid PMTCT Cohort), Pablo Rojo (CORISPES‐Madrid), Antoni Soriano‐Arandes (NENEXP), Manuel Battegay (SHCS), Roger Kouyos (SHCS), Cristina Mussini (Modena Cohort), Pat Tookey (NSHPC), Jordi Casabona (PISCIS), José M. Miró (PISCIS), Antonella Castagna (San Raffaele), Deborah Konopnick (St. Pierre Cohort), Tessa Goetghebuer (St Pierre Paediatric Cohort), Anders Sönnerborg (Swedish InfCare), Carlo Torti (MODENA), Caroline Sabin (UK CHIC), Ramon Teira (VACH), Myriam Garrido (VACH) and David Haerry (European AIDS Treatment Group).

Executive Committee: Stéphane de Wit (Chair, St. Pierre University Hospital), Jose Mª Miró (PISCIS), Dominique Costagliola (FHDH), Antonella d'Arminio‐Monforte (ICONA), Antonella Castagna (San Raffaele), Julia del Amo (CoRIS), Amanda Mocroft (EuroSida), Dorthe Raben (Head, Copenhagen Regional Coordinating Centre) and Geneviève Chêne (Head, Bordeaux Regional Coordinating Centre). Paediatric Cohort Representatives: Ali Judd and Pablo Rojo Conejo.

Regional Coordinating Centres: Bordeaux RCC: Diana Barger, Christine Schwimmer, Monique Termote and Linda Wittkop; Copenhagen RCC: Maria Campbell, Casper M. Frederiksen, Nina Friis‐Møller, Jesper Kjaer, Dorthe Raben and Rikke Salbøl Brandt.

Project Leads and Statisticians: Juan Berenguer, Julia Bohlius, Vincent Bouteloup, Heiner Bucher, Alessandro Cozzi‐Lepri, François Dabis, Antonella d'Arminio Monforte, Mary‐Ann Davies, Julia del Amo, Maria Dorrucci, David Dunn, Matthias Egger, Hansjakob Furrer, Marguerite Guiguet, Sophie Grabar, Ali Judd, Ole Kirk, Olivier Lambotte, Valériane Leroy, Sara Lodi, Sophie Matheron, Laurence Meyer, Jose Mª Miró, Amanda Mocroft, Susana Monge, Fumiyo Nakagawa, Roger Paredes, Andrew Phillips, Massimo Puoti, Michael Schomaker, Colette Smit, Jonathan Sterne, Rodolphe Thiebaut, Claire Thorne, Carlo Torti, Marc van der Valk, Linda Wittkop and Natasha Wyss.
