# Supplementary material for: Higher rates of triple‐class virological failure in perinatally HIV‐infected teenagers compared with heterosexually infected young adults in Europe
Source: HIV Med. 2016 Sep 14;18(3):171–80. doi: 10.1111/hiv.12411 (PMC5298034; doi:10.1111/hiv.12411)
Supplement: Supplementary file 1 — Table S1. Number and proportion of participants developing virological failure, interrupting treatment, and developing TCVF, by risk group and age at the start of ART. Table S2. Outcomes of TCVF, by risk group and age at the start of ART. [file HIV-18-171-s001.docx]

**Supplementary Table 1.** Number and proportion of participants developing virological failure, interrupting treatment, and developing TCVF, by risk group and age at the start of ART

| Risk group | Perinatal | | | | | | Heterosexual | | | |
| --- | --- | --- | --- | --- | --- | --- | --- | --- | --- | --- |
| Age in years at start of ART | < 2 | 2 - 4 | 5 - 9 | 10 - 14 | 15 - 19 | *All* | 15 - 19 | 20 - 24 | 25 - 29 | *All* |
| Number of participants | 238 | 148 | 229 | 171 | 20 | *806* | 264 | 1459 | 3443 | *5166* |
| Follow-up from ART start *pyrs*  Median  (IQR) | 4.9  (2.7-7.0) | 5.1  (3.3-6.9) | 4.5  (2.7-6.2) | 3.0  (1.5-4.3) | 1.4  (1.0-2.3) | *4.1*  *(2.3-6.1)* | 3.5  (1.8-5.7) | 3.3  (1.6-5.8) | 3.4  (1.7-5.8) | *3.4*  *(1.7-5.8)* |
|  |  |  |  |  |  |  |  |  |  |  |
| No. (%) who developed VF | 119  (50.0%) | 63  (42.6%) | 99  (43.2%) | 70  (40.9%) | 6  (30.0%) | *357*  *(44.3%)* | 100  (37.9%) | 531  (36.4%) | 1143  (33.2%) | *1774*  *(34.3%)* |
| Estimated cumulative % who had VF by  3 yrs  (95% CI)  5 yrs  (95% CI) | 48.9  (42.2-55.6)  54.0  (46.9-61.1) | 35.7  (27.7-43.7)  44.9  (36.0-53.8) | 33.6  (27.1-40.2)  47.3  (39.7-54.9) | 44.3  (36.0-52.7)  51.1  (41.2-60.9) | - | *40.7*  *(37.0-44.3)*  *49.9*  *(45.8-54.0)* | 34.0  (27.9-40.1)  44.8  (37.4-52.2) | 34.0  (31.3-36.7)  45.9  (42.6-49.2) | 32.0  (30.3-33.7)  39.0  (37.0-41.0) | *32.7*  *(31.2-34.1)*  *41.2*  *(39.5-42.8)* |
| No. (%) who developed TCVF | 18  (7.6%) | 11  (7.4%) | 21  (9.1%) | 20  (11.7%) | 0  (0%) | *70*  *(8.7%)* | 7  (2.7%) | 61  (4.2%) | 114  (3.3%) | *182*  *(3.5%)* |
| Estimated cumulative % with TCVF by  3 yrs  (95% CI)  5 yrs  (95% CI) | 3.7  (1.0-6.4)  7.8  (3.7-12.0) | 3.4  (0.1-6.6)  4.5  (0.6-8.5) | 3.6  (1.0-6.2)  8.4  (3.9-12.9) | 9.8  (4.5-15.0)  27.7  (13.2-42.1) | - | *4.7*  *(3.1-6.4)*  *9.6*  *(7.0-12.3)* | 2.1  (0.0-4.1)  2.1  (0.0-4.1) | 2.3  (1.3-3.2)  5.8  (4.0-7.6) | 1.9  (1.3-2.4)  4.5  (3.5-5.4) | *2.0*  *(1.5-2.5)*  *4.7*  *(3.9-5.5)* |
| Number of treatment interruptions | 81 | 38 | 70 | 40 | 7 | *236* | 133 | 580 | 1088 | *1801* |
| Rate of interruption  per 100 person-years  (95% CI) | 8.0  (6.3-9.9) | 5.4  (3.8-7.4) | 7.4  (5.7-9.3) | 8.4  (6.0-11.4) | - | *7.4*  *(6.5-8.4)* | 15.4  (12.9-18.2) | 11.6  (10.7-12.6) | 8.7  (8.2-9.3) | *9.8*  *(9.4-10.3)* |
| Number of restarts after interruption | 51 | 27 | 44 | 27 | 6 | *155* | 100 | 431 | 846 | *1377* |
| Rate of restarting  per 100 person-years  (95% CI) | 66.2  (49.3-87.1) | 46.6  (30.7-67.7) | 115.8  (84.1-155.4) | 49.1  (32.4-71.4) | - | *67.4*  *(57.2-78.9)* | 65.4  (53.2-79.5) | 62.9  (57.1-69.1) | 52.7  (49.2-56.4) | *56.4*  *(53.5-59.5)* |

**Supplementary Table 2:** Outcomes of TCVF, by risk group and age at the start of ART

| Risk group | Perinatal | | | | | Heterosexual | | | |
| --- | --- | --- | --- | --- | --- | --- | --- | --- | --- |
| Age in years at start of ART | < 2 | 2 - 4 | 5 - 9 | 10 – 14 | *All* | 15 - 19 | 20 - 24 | 25 - 29 | *All* |
| Number who  developed TCVF | 18 | 11 | 21 | 20 | *70* | 7 | 61 | 114 | *182* |
| Number who have  follow-up after TCVF | 18 | 10 | 19 | 17 | *64* | 6 | 58 | 106 | *170* |
| One year after TCVF  *median (IQR)*  CD4 count *cells/mm^3^*  CD4 percentage  Viral load *log_10_ c/ml*  Viral load on ART *log_10_ c/ml* | 1130  (754-1285)  *n=13*  28  (22-35)  *n=12*  3.1  (2.0-3.6)  *n=12*  3.6  (3.5-3.7)  *n=5* | 509  (385-920)  *n=10*  26  (15-30)  *n=10*  3.1  (1.7-4.5)  *n=10*  1.7  (1.7-3.9)  *n=6* | 477  (239-630)  *n=15*  18  (13-25)  *n=15*  3.6  (1.7-3.9)  *n=15*  3.5  (1.7-3.7)  *n=9* | 162  (73-288)  *n=9*  6  (5-12)  *n=9*  4.4  (4.4-5.2)  *n=9*  4.4  (1.7-4.4)  *n=5* | *540*  *(239-920)*  *n=47*  *22*  *(12-28)*  *n=46*  *3.6*  *(1.7-4.5)*  *n=46*  *3.5*  *(1.7-3.9)*  *n=25* | 363  (242-418)  *n=5*  -  3.2  (3.1-3.4)  *n=5*  3.1  (2.4-3.3)  *n=4* | 286  (190-475)  *n=47*  -  2.3  (1.7-3.9)  *n=46*  2.7  (1.7-4.1)  *n=27* | 334  (183-478)  *n=67*  -  2.7  (1.7-4.3)  *n=70*  2.7  (1.7-4.2)  *n=46* | *305*  *(190-475)*  *n=119*  *-*  *2.6*  *(1.7-4.2)*  *n=121*  *2.7*  *(1.7-4.1)*  *n=77* |
| AIDS diagnosis after TCVF | 1 | 0 | 1 | 0 | *2* | 3 | 3 | 10 | *16* |
| Death after TCVF | 0 | 0 | 0 | 1 | *1* | 0 | 1 | 2 | *3* |
| Treatment change by one year after TCVF | 3 | 1 | 2 | 0 | *6* | 3 | 14 | 26 | *43* |
